# Copy Number Heterogeneity of JC Virus Standards

**DOI:** 10.1128/JCM.02337-16

**Published:** 2017-02-22

**Authors:** Alexander L. Greninger, Allen C. Bateman, Ederlyn E. Atienza, Sharon Wendt, Negar Makhsous, Keith R. Jerome, Linda Cook

**Affiliations:** a Department of Laboratory Medicine, University of Washington, Seattle, Washington, USA; b Fred Hutchinson Cancer Research Institute, Seattle, Washington, USA; Memorial Sloan-Kettering Cancer Center

**Keywords:** BKV, JC virus, T antigen, clinical standards, deep sequencing, polyomavirus, qPCR, simian virus 40

## Abstract

Quantitative PCR is a diagnostic mainstay of clinical virology, and accurate quantitation of viral load among labs requires the use of international standards. However, the use of multiple passages of viral isolates to obtain sufficient material for international standards may result in genomic changes that complicate their use as quantitative standards. We performed next-generation sequencing to obtain single-nucleotide resolution and relative copy number of JC virus (JCV) clinical standards. Strikingly, the WHO international standard and the Exact v1/v2 prototype standards for JCV showed 8-fold and 4-fold variation in genomic coverage between different loci in the viral genome, respectively, due to large deletions in the large T antigen region. Intriguingly, several of the JCV standards sequenced in this study with large T antigen deletions were cultured in cell lines immortalized using simian virus 40 (SV40) T antigen, suggesting the possibility of transcomplementation in cell culture. Using a cutoff 5% allele fraction for junctional reads, 7 different rearrangements were present in the JC virus sequences present in the WHO standard across multiple library preparations and sequencing runs. Neither the copy number differences nor the rearrangements were observed in a clinical sample with a high copy number of JCV or a plasmid control. These results were also confirmed by the quantitative real-time PCR (qPCR), droplet digital PCR (ddPCR), and Sanger sequencing of multiple rearrangements. In summary, targeting different regions of the same international standard can result in up to an 8-fold difference in quantitation. We recommend the use of next-generation sequencing to validate standards in clinical virology.

## INTRODUCTION

Polyomaviruses are unenveloped, double-stranded DNA viruses with a circular genome (1). Despite the discovery of multiple polyomaviruses over the last decade, only four polyomaviruses have been associated with human disease (1–5). JC virus (JCV) infection has been associated with progressive multifocal leukoencephalopathy (PML) in immunosuppressed patients (6, 7). JC virus has also been associated with two other neurological diseases, JC virus granule cell neuronopathy and JC virus encephalopathy (7).

Detection of JCV in the cerebrospinal fluid (CSF) is required for the diagnosis of PML, and quantitative levels of the virus have been associated with clinical outcomes of PML patients, with lower DNA values associated with better outcomes (8–11). However, viral loads in patients can vary dramatically, meaning analytically sensitive assays are required to protect against false-negative tests (12). Real-time PCR is commonly used to measure levels of JCV in CSF (13).

Quantitative standards are of critical importance to real-time PCR in order to quantitate copies and compare different assays (14). The creation of laboratory standards in virology often relies on growing up large amounts of virus in cell culture to recapitulate the viral particle in extraction and to ensure future preparations are not required. However, passaging a virus several times can create selection pressures that do not recapitulate viral biology *in vivo*. In this study, we used next-generation sequencing and confirmatory quantitative real-time PCR (qPCR) and droplet digital PCR (ddPCR) to show that several of the standards used for the JCV quantitative PCR mixture contain multiple rearrangements, some with large deletions in the T antigen region, that incur large variability in measured copies depending on the locus measured. We hypothesize that these deletions are due to passage of the virus in simian virus 40 (SV40) T antigen immortalized cell lines.

## RESULTS

**FIG 1:**
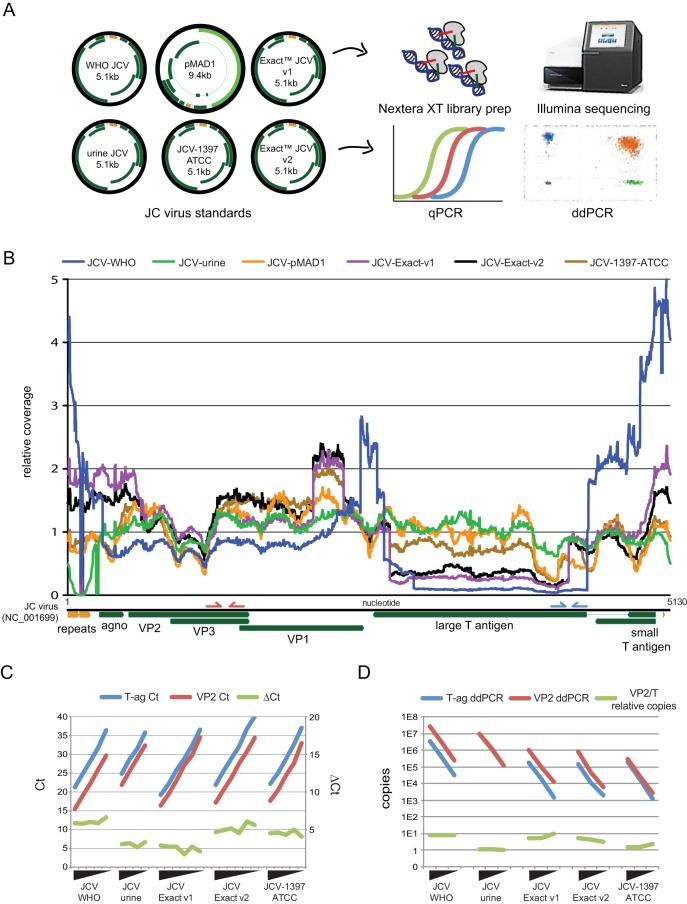
Next-generation sequencing of JC virus standards reveals deletions and copy number heterogeneity. (A) Six different JC virus materials were deep sequenced, and five standards were tested by qPCR and ddPCR. Gene organization of each JC virus is shown in green, with regulatory regions depicted in orange. Of note, the 9.4-kb pMAD1 plasmid inserted the backbone at the location of the probe used in the T antigen qPCR and ddPCR assay and cannot be quantitated at that locus. (B) Coverage plot of six different standards of JC virus mapped to the JC virus NCBI reference genome (GenBank accession number NC_001699). The *y* axis is normalized such that 1 designates the average coverage across the viral genome to highlight relative differences in coverage. Three of the six standards include a large deletion in the T antigen region that constitutes a greater than 4-fold difference in copy number relative to the structural genes. Reductions in coverage in the regulatory repeat region are due both to small deletions and sequence divergence relative to JC virus reference genome. Primers for the Focus PCR analyte-specific reagent targeting the VP2/3 region are shown on the JC virus genome in red, while the pep primers targeting the T antigen region are shown in blue. (C, D) Confirmation of the copy number differences seen by sequencing was performed with qPCR (C) and ddPCR (D) using PCR primers against the VP2/3 gene (red) and T-ag gene (blue). Ten-fold dilutions of each of the standards depicted were quantitated, and the discrepancy in cycle threshold (*C_T_*) and absolute quantitation (D) between the VP2/3 and T-ag assays are depicted (green) for each standard. Δ*C_T_*, change in *C_T_*.

Based on our previous discovery of copy number heterogeneity in the BK virus international standard, we performed next-generation sequencing on a variety of JCV standards present in our laboratory. We obtained a provisional WHO standard for JCV, two different versions of the Exact (v1 and v2) standard, a urine specimen with a high copy number of JCV present, the ATCC 1397 JC virus strain, and the original 1980 plasmid with the Mad-1 strain of JC virus cloned into it. All strains were sequenced using Nextera XT libraries on an Illumina MiSeq. qPCR and ddPCR using the Focus PCR primers targeting the VP2/3 region and the University of Washington (UW) Virology clinical primers targeting the T antigen region were performed as well (refer to Fig. 1B for primers).

Deep sequencing of the JC virus standards resulted in between 0.4% and 80% of the sequencing reads mapping to the JC virus (Table 1). Sequencing of DNA extracted from a urine specimen that was known to contain a high copy number of JC virus as well as DNA extracted from a plasmid containing cloned JC virus both yielded the lowest variation in coverage (Fig. 1B). qPCR and ddPCR quantitation of the clinical urine specimen indicated equal copy numbers at the VP2/3 and T antigen loci of these materials.

**TABLE 1:** Next-generation sequencing results of JC virus standards

Strain	Total reads	JC virus reads	Mean coverage
WHO	512,609	406,100	13,445
JCV urine	299,886	59,115	1,574
pMAD1 plasmid	2,460,512	963,917	26,060
Exact v1	586,222	96,765	2,943
Exact v2	2,602,925	9,860	383
ATCC 1397	1,694,258	11,527	393

**FIG 2:**
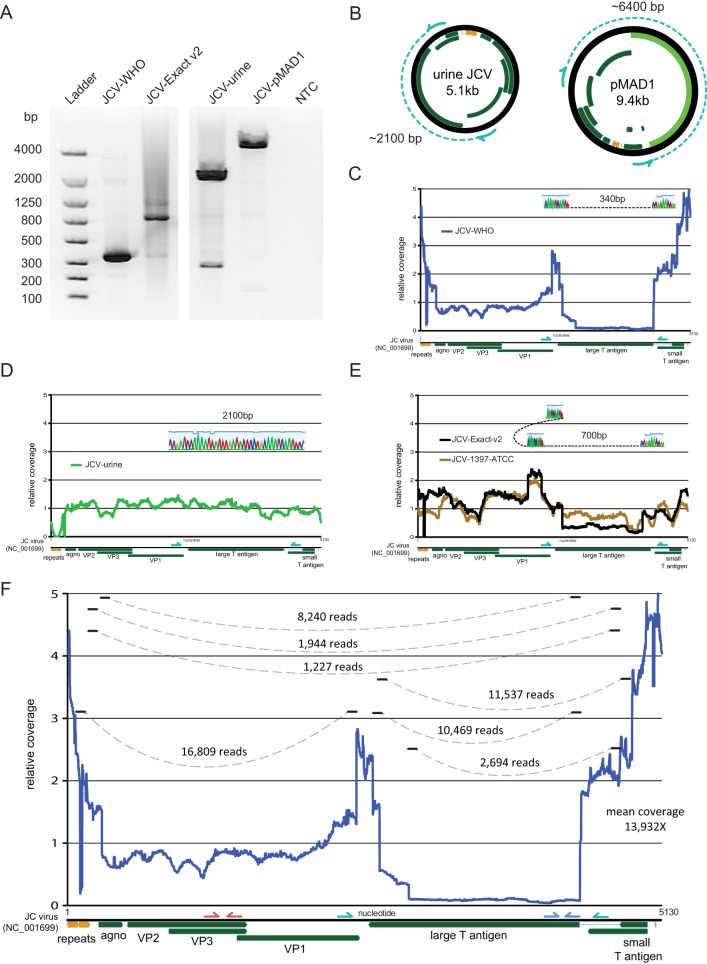
Sanger confirmation of junction reads from next-generation sequencing data. (A) Gel electrophoresis of PCR products amplified with primers between nucleotides 2416 and 4543 based on the JC virus reference genome in NCBI (GenBank accession number NC_001699). NTC, no template control. (B) Expected PCR amplicons in control materials used in this study based on nucleotide distance. The JC virus plasmid pMAD1 contains a 4-kb backbone insert within this PCR amplicon. (C) The PCR amplicon of 340 bp recovered from the WHO standard demonstrates one of the large deletions in the T antigen region that was first identified by next-generation sequencing data. (D) The PCR amplicon of 2,100 bp demonstrates no deletion in the T antigen region in a JC virus from a clinical urine specimen. (E) The PCR amplicon of 700 bp demonstrates a complex rearrangement in the Exact v2 standard and JCV ATCC 1397 strain that was first identified by next-generation sequencing data. (F) Junctional reads with more than 5% allele frequency from the deep sequencing of the WHO JC virus standard are depicted.

The WHO international standard contained an approximate 8-fold variation in coverage between structural genes and much of the T antigen region and contained the largest coefficient of variation in coverage among the strains at 98.4% (Fig. 1B and D). Analysis of junctional reads present in the WHO international standard at a more than 5% allele frequency revealed 7 different rearrangements between loci more than 50 bp apart. The Exact v1 and v2 standards and the ATCC 1397 standard contained a complex rearrangement that resulted in the same duplication of the C terminus of the VP2/3 gene inserted into a C terminus of the T antigen (Fig. 2). Multiple rearrangements in these strains that were detected by deep sequencing were confirmed by PCR and Sanger sequencing (Fig. 2). Of note, both of the Exact strains as well as the ATCC 1397 strain were grown in Cos cell lines (J. Boonyarantanakornkit, Exact Diagnostics, personal communication). The Exact v1 and v2 standards both yielded an approximate 4- to 5-fold difference in copy number between structural genes and T antigen, while the ATCC 1397 strain had a 1.5-fold difference in copy number between these loci (Fig. 1C and D; see also Table S1 in the supplemental material).

## DISCUSSION

Using deep sequencing and confirmatory qPCR, ddPCR, and Sanger sequencing, we show four different qPCR standards for strains containing JC virus with large, different deletions in the T antigen region. These deletions result in significant copy number differences between different loci in the JC virus genome that may lead to radically different sensitivities and quantities being reported by various JC virus clinical qPCR assays if these standards are adopted by the clinical virology community. In contrast, deep sequencing of a clinical specimen with JC virus and of a plasmid containing the JC virus sequence do not show the large differences in copy number found in the cell culture-adapted strains. These results mirror those we recently reported in an international standard for BK virus (41).

Of note, most of the JC virus standards sequenced in this study were cultured in the Cos cell line. The Cos cell line is derived from a primary monkey cell line that was transformed with SV40 T antigen and expresses both large T antigen and small t antigen (15). Given that the deletions seen in this study occurred in the T antigen region of the JC virus, that they were seen in approximately 90% of the JC virus strains present, and that T antigen is known to be required for polyomavirus replication, we hypothesize that the SV40 T antigen in the Cos cell line is providing nonstructural functions, such as DNA binding, unwinding, viral replication, and transformation in *trans*, to the JC virus strains with the T antigen deletions sequenced in this study. Indeed, SV40 T antigen has been shown to be required for archetype JC virus replication in Cos cells, as JC virus is unable to replicate in the untransformed Cos parental cell line CV-1 (16). The presence of SV40 T antigen also allows primary human fetal glial cells to support the growth of JC virus, which otherwise does not grow (17). Thus, SV40 T-antigen-transformed cell lines may have been specifically chosen for their ability to support high levels of JC virus growth given the need for large amounts of highly concentrated virus to provide qPCR standards to multiple labs around the world, albeit with the unforeseen consequence that the cell line would yield JC virus strains with large deletions.

The many functions of the polyomavirus large T antigen include binding multiple host proteins (including p68 DNA polymerase/primase, p53, Rb, hsc70, and replication protein A), binding to itself to form hexamer, binding the origin of replication, unwinding DNA, and translocating to the nucleus (18, 19). Of these, only origin binding would be potentially compromised by complementation with a different origin. The BK, JC, and SV40 viruses have nearly identical sequences in their origin of replication and surrounding sequences (20). They only differ in the singular nucleotide flanking the pentanucleotide GAGGC motif that is not involved in hairpin formation (SV40, gaggcCgaggc; JCV, gaggcGgaggc; BKV, gaggcAgaggc) (1). Previous experiments with SV40 T antigen have shown that it is capable of binding to the JC virus and BK virus origin sequence and initiating replication *in vitro* and *in vivo* (21, 22). Indeed, the SV40 T antigen is noted to have an even broader sequence-binding capability than that of JC virus (23).

The recovery of the JC and BK virus sequences with large deletions in cell culture is likely due to the unique biology and sequence identity of the polyomaviruses. Encapsidation of viral DNA is thought to be dependent on viral structural proteins with no contribution from viral nonstructural proteins beyond DNA replication (24). The amino acid sequence conservation between SV40 and the JC and BK virus T antigen is approximately 73%. As described above, the origin DNA sequence is nearly identical between different polyomaviruses, with the nucleotide sequence required for T antigen binding being absolutely conserved. The JC virus T antigen J domain can replace the SV40 J domain and retain replication activity (25). Chimeras of JC virus and SV40 showed that viruses containing JC virus regulatory sequences and SV40 coding regions can replicate, which is consistent with the JC virus recovered in this study (26). Thus, polyomavirus nonstructural proteins show the ability to complement each other (27, 28). Indeed, different deletions were recovered in three of the cell culture-associated BK and JC polyomaviruses sequenced, suggesting that each of these deletions arose independently in culture.

Our study shows the importance of deep sequencing standards to validate reagent integrity before they are scaled internationally (29). This study adds to the many uses of next-generation sequencing in the clinical virology laboratory (30, 31). The main limitation of our study is the use of short-read sequencing to sequence the strain, as we are thus unable to link rearrangements across the multiple viruses present in each standard (32). Deep sequencing provides single-nucleotide resolution of the sequences present and the relative copy numbers of loci across the genome. Deep sequencing of these standards demonstrated the presence of multiple viral species with radically different copy numbers due to deletions, as well as single-nucleotide changes that may affect PCR primer binding and overall quantitation, making growth of BK and JC viruses in SV40-transformed cell lines a potentially suboptimal choice for a qPCR international standard.

## MATERIALS AND METHODS

### JC virus materials.

The 1st WHO international standard for JC virus DNA (National Institute for Biological Standards and Control [NIBSC] code 14/114) was reconstituted in 1 ml of nuclease-free molecular-grade water and left for 20 min with occasional gentle agitation before use according to recommendations. The recommendations then instruct the dilution of the international standard in the matrix routinely used within the laboratory for clinical diagnosis of JCV DNA and that the diluted material should be extracted prior to JCV DNA measurement. We diluted the international standard 1:1 into 1 ml of normal serum control (NSC; Bio-Rad) followed by serial 10-fold dilutions in NSC prior to extraction.

The Exact Diagnostics (Fort Worth, TX) JCV prototype panel v1 and JCV prototype panel v2 each consist of six concentrations (1 × 10^7^, 1 × 10^6^, 1 × 10^5^, 1 × 10^4^, 1 × 10^3^, and 1 × 10^2^ copies/ml) of whole, intact JC virus strain MAD-4. Each concentration was extracted and run on qPCR and ddPCR as described below.

JC virus strain ATCC 1397 was diluted for use as an in-house JCV-positive control. Control material was serially diluted 10-fold in NSC prior to extraction for ddPCR. For qPCR, the highest concentration was extracted and serial 10-fold dilutions were made in 10 mM Tris-HCl.

A JCV-positive urine sample was serially diluted 10-fold in NSC prior to extraction and run on qPCR and ddPCR as described below.

Advanced Biotechnologies Inc. (Eldersburg, MD) JC human polyomavirus (MAD1 strain) viral qDNA PCR control (17-943-500) was obtained at a stated concentration of 5 × 10^6^ copies/ml of DNA. This material was serially diluted 10-fold in 10 mM Tris and run on qPCR and ddPCR without extraction as described below.

A plasmid containing the MAD-1 strain was ordered from Addgene (plasmid 25626), miniprepped, and diluted in Tris-EDTA (TE) buffer to approximately 10^6^ copies/ml (33). This material was then serially diluted 10-fold in 10 mM Tris and run on qPCR and ddPCR without extraction as described below. As the plasmid backbone insert disrupts the T antigen probe-binding site, the plasmid was most useful as a sequencing control.

### DNA extraction and quantitative real-time PCR.

The Roche MagNa Pure 96 (Roche, Indianapolis, IN) was used to extract DNA from the WHO, Exact v1 and v2, ATCC 1397, and urine materials according to manufacturer recommendations. The input volume was 500 μl, and the elution volume was 100 μl. Extracted DNA was either used immediately or stored for up to 24 h at 4°C before PCR.

The quantitative real-time PCR assay used was the clinical JCV assay that is currently run at the University of Washington Clinical Virology (34, 35). This assay targets the large T region with a single primer pair (PEP1, AGTCTTTAGGGTCTTCTACC; PEP2, GGTGCCAACCTATGGAACAG) and a 6-carboxyfluorescein (FAM)-6-carboxytetramethylrhodamine (TAMRA) probe (FAM-TGATGATGAAAACACAGGATCCCAACACTC-TAM). The qPCR is performed in the Bio-Rad SsoAdvanced Universal Probes Supermix at 50°C for 2 min, 95°C for 2 min, and 45 cycles of 95°C for 20 s and 60°C for 1 min. Real-time quantification was also performed using the JCV primer pair (MOL9021, analyte-specific reagent [ASR] marked) and the 2.5× universal master mix from Focus Diagnostics (DiaSorin, Cypress, CA) on the Focus integrated cycler under standard cycling conditions. The primer pair includes a Scorpion primer/probe and targets a conserved region of the VP2/3 gene as described below. WHO dilutions were used to convert quantification cycle (*C_q_*) to copies per milliliter in post amplification analysis.

### Droplet digital PCR.

Prior to ddPCR, restriction enzyme digestion was performed with HindIII-HF (New England BioLabs, Ipswitch, NY). To 5 μl of extracted DNA, 3 μl of water, 1 μl of 10× CutSmart buffer, and 1 μl of HindIII-HF (20,000 U/ml) were added followed by incubation at 37°C for 1 h. After incubation, the mixture was diluted 1:5 by the addition of 40 μl of water, and 10 μl of this dilution was used per PCR.

Droplet digital PCR was performed using the Bio-Rad system. Bio-Rad ddPCR mastermix, primers, and probes (final primer and probe concentrations equal to final qPCR concentrations) were added to a 96-well plate and vortexed, followed by droplet generation on the QX100 droplet generator. Droplets were transferred to a 96-well PCR plate and amplified on a 2720 Thermal Cycler (Applied Biosystems) with the following thermocycling parameters: 94°C for 10 min, followed by 40 cycles of 94°C for 30 s and 60°C for 1 min, and a 98°C hold for 10 min. Droplets were read in the QX200 droplet reader (Bio-Rad) immediately following amplification. Data were analyzed with QuantaSoft analysis v1.3.2.0, and quantification was calculated to reflect copies per milliliter of the initial specimen.

### Next-generation sequencing.

Extracted DNA from the standards was diluted to 0.1 to 0.2 ng/μl and was used for dual-indexed Nextera XT sequencing library preparation followed by 18 cycles of amplification (36). Strains from the WHO, Exact v1, pMAD1 plasmid, as well as a clinical strain from urine were sequenced on a single-end 185-bp run on an Illumina MiSeq. JCV 1397 from the ATCC, WHO, Exact v1, Exact v2, and the urine clinical strain were also sequenced on a 2 × 260-bp run on an Illumina MiSeq. Sequencing reads were adapter and quality filtered (Q30) using cutadapt and aligned to the JC virus reference genome (GenBank accession number NC_001699) using the Geneious v9.1.4 mapper with structural variant detection enabled (37–39). Coverage maps were produced from bam files generated using Geneious using the genomecov option from bedtools (40).

To determine the locus used in the Focus Diagnostics analyte-specific reagent, an amplicon created via real-time PCR with the Focus primers was subjected to half reaction of end repair and dA tailing, followed by adapter ligation and PCR amplification with TruSeq adapters using a Kapa HyperPlus kit. The indexed amplicon was sequenced on a 2 × 500-bp MiSeq run, and reads were adapter/quality-trimmed and mapped to the JC virus reference genome as above. The location of the primers is depicted in Fig. 1B.

### Confirmatory Sanger sequencing.

Extracted DNA from the standards was subjected to 35 cycles of PCR amplification in a 20-μl total volume with 14.5 μl of water, 4 μl of 5× HF buffer, 0.5 μl of 12.5 mM deoxynucleoside triphosphates (dNTPs), 0.5 μl of Phusion polymerase (Thermo), and 0.5 μl each of 10 μM primers JCV-2416F (5′-CATGGATGCTCAAGTAGAGG-3′) and JCV-4543R (5′-CTGGGAGAAAGTTCTTGGAG-3′). PCR products were visualized on a 1.2% FlashGel (Lonza) and extracted from a 1.5% agarose-LB (Faster Better Media) using a Zymoclean gel DNA recovery kit and submitted for bidirectional Sanger sequencing.

## Supplementary Material

Supplemental material
