# Supplementary material for: Copy Number Heterogeneity of JC Virus Standards
Source: J Clin Microbiol. 2017 Feb 22;55(3):824–31. doi: 10.1128/JCM.02337-16 (PMC5328450; doi:10.1128/JCM.02337-16)
Supplement: Supplemental material [file supp_55_3_824__index.html]

Copy Number Heterogeneity of JC Virus Standards — Supplemental material 

# Copy Number Heterogeneity of JC Virus Standards

## Supplemental material

- Supplemental file 1 -

  Table S1 (qPCR and ddPCR data depicted in Fig. 1C and D)

  XLSX, 45K
